# Coronavirus (COVID-19) in Italy: knowledge, management of patients and clinical experience of Italian dentists during the spread of contagion

**DOI:** 10.1186/s12903-020-01187-3

**Published:** 2020-07-10

**Authors:** Alessandra Putrino, Mario Raso, Cosimo Magazzino, Gabriella Galluccio

**Affiliations:** 1 Advanced Training Course in Risk Management in Healthcare and Professional Responsibility, University “Sapienza” of Rome, Piazzale Aldo Moro 5, 00185 Rome, Italy; 2 Italian Society for Applied and Industrial Mathematics (SIMAI), Rome, Italy; 3 Department of Political Sciences, University “Roma Tre”, Rome, Italy; 4 Department of Oral and Maxillofacial Sciences, University “Sapienza”, Rome, Italy

**Keywords:** 2019 novel coronavirus, COVID-19, Dentistry, Health management, Knowledge, Survey

## Abstract

**Background:**

The coronavirus infection that emerged in China in the last few months of 2019 has now spread globally. Italy registered its first case in the second half of February, and in a short time period, it became the top country in Europe in terms of the number of infected people and the first in the world in terms of deaths. The medical and scientific community has been called upon to manage the emergency and to take measures. Dentists also need to take new precautions during their clinical activity to protect themselves, coworkers and patients from the risks of contagion and to avoid further spread of infection.

**Methods:**

Following the data published in the international literature as well as the guidelines and directives constantly updated by the WHO and by the national health authorities, a questionnaire to be completed anonymously was submitted online to Italian dentists using social tools and online professional platforms. The collected data were processed statistically, providing descriptive data and analysis of correlations of the most significant parameters using the Pearson’s χ2, the Likelihood-Ratio χ2, Cramér’s V, Fisher’s exact test, Goodman and Kruskal’s γ, and Kendall’s τb (*p* < 0.05).

**Results:**

A total of 535 dentists from Italy participated in the survey. A good level of scientific knowledge about coronavirus and the extra precautionary measures needed to limit the spread was related to the age of respondents and their sex. Coming from areas with higher concentrations of cases affected knowledge, level of attention and perception of risk related to dental activity.

**Conclusions:**

At the moment, there are no therapies or vaccines to contain the infection with the new coronavirus that is causing many infections, many of which are fatal, worldwide. Dentists are one of the categories at highest risk of encountering diseases and infections because they work in close proximity with patients, and in their procedures, there is always contact with aerosols with high bacterial and viral potential. Therefore, during this COVID-19 emergency, it is important that dentists are properly informed and take the appropriate precautionary measures.

## Background

**Fig. 1:**
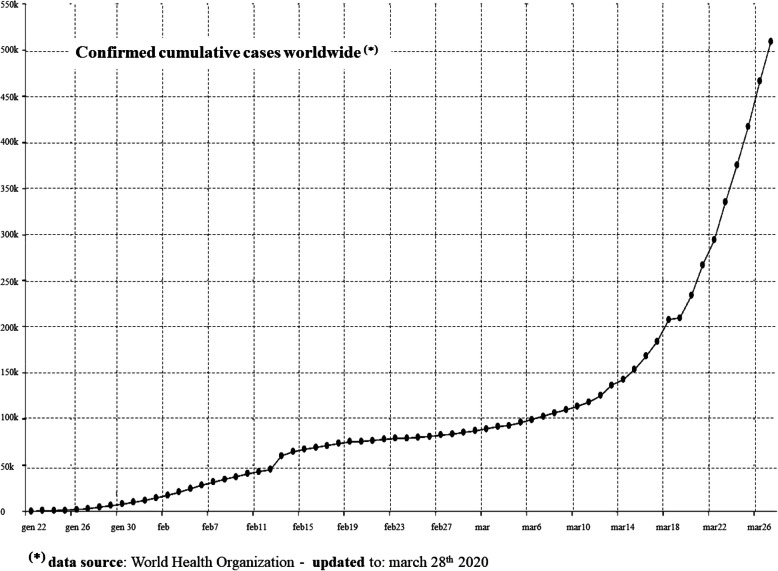
Confirmed cumulative cases. Data Source: World Health Organization. Updated to March 28th 2020

**Fig. 2:**
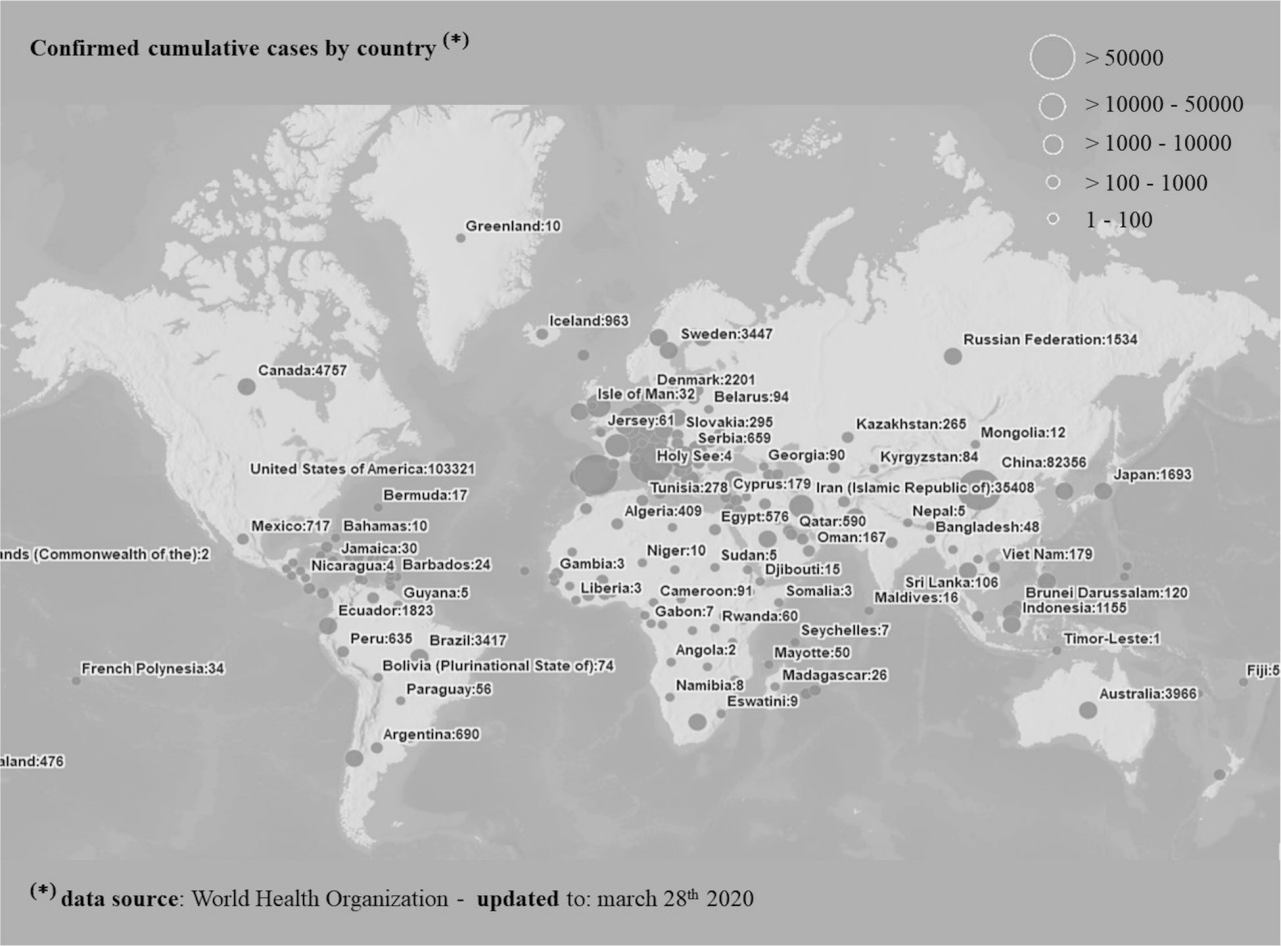
Confirmed cumulative cases by country. Data Source: World Health Organization. Updated to March 28th 2020

Human coronaviruses are a group of RNA viruses able to cause respiratory, gastrointestinal and central nervous system diseases. The first human coronavirus (HCoV) was detected in the mid-1960s [1]. In December 2019, the seventh coronavirus known to infect humans was found in China (Wuhan city, Hubei Province) [2]. On 12 January 2020, the World Health Organization (WHO) announced the temporarily named nCoV-2019, now called SARS-CoV-2, as the novel coronavirus pathogen responsible for the increasing number of new pneumonia cases [3, 4]. Since that day, the number of cases in China and progressively in many other parts of the world has increased (Fig. 1), and with it, the number of people who died because of this infection as the primary or contributory cause of preexisting illness [5]. On 11 March 2020, the WHO stated that the novel coronavirus outbreak was a pandemic (Fig. 2). The scientific community is still making numerous efforts to clarify the etiology, pathogenicity, and characteristics of the virus to establish the mechanisms underlying human-to-human transmission and possible treatments [6–10]. Although there are many cases of recovery, the number of deceased subjects has increased with the spread, particularly affecting older subjects with previous severe pathologies [11, 12]. The first detection of the virus in Italy was in a case of two Chinese tourists from Wuhan who were later treated at the Spallanzani Hospital of Rome. The entire tourist group of the Chinese couple was then quarantined for two weeks in the same hospital and then released after negative results of the clinical and serologic controls [13].

**Fig. 3:**
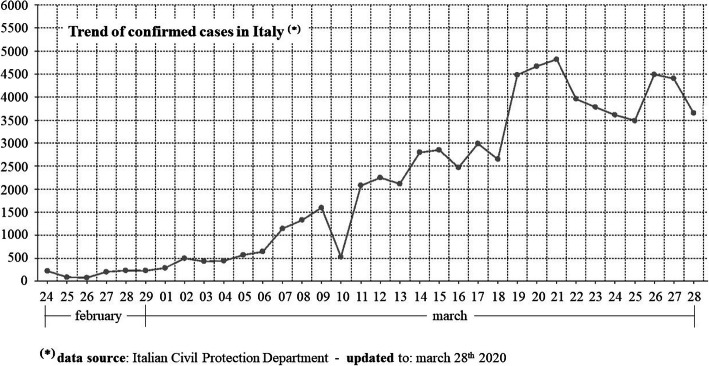
Trend of confirmed cases in Italy. Data Source: Italian Civil Protection Department. Updated to 28th March 2020

On 21 February, the first locally transmitted case, a 38-year-old man who never travelled in China, emerged in northern Italy, Lombardy region, Codogno town, and since then, the number of cases has increased (Fig. 3) in the whole area and progressively increased in many other northern regions until the virus has spread to affect the entire Italian territory. As the number of swab tests for suspected cases has continued to increase, the Italian authorities have started to adopt preventive measures to try to isolate the affected areas and block the spread of the infections and a nationwide lockdown went into effect on 10 March. The death toll, at the moment, has reached 10,023 persons (mostly elderly people with other preexisting diseases), and the number of infected cases is greater than 70,065, with 12,384 recovered (Table 1). Italian government, the Ministry of Health, Civil Protection and other competent bodies at the local level are constantly engaging in this emergency, providing instructions to citizens and health workers and updating the population on the evolution of the situation [14–17]. Even dentists have been involved in the management of this emergency through indications on prevention and safety measures to be observed in their clinical activity due to the high level of exposure for operators and dental patients. In the last weeks the number of health workers infected has risen: many nurses and doctors on the coronavirus front lines are working without adequate personal protective equipment (PPE), exposing themselves to great risk and some of them have been infected while on duty. Already, 50 doctors have died, and 4 of them were dentists [18]. A direct correlation between their death and coronavirus infection was not, however, ascertained but many of them were engaged in the management of infections. The novel coronavirus was recently identified in the saliva of infected patients. Dental clinical procedures generate droplets and aerosols that can lead to viral transmission [19]. Contamination on surfaces and diffusion by contact, conceivable due to the characteristics of dental activity, require a great deal of attention from dentists, who should adopt simple but effective practical strategies to stop the possible spread of the virus. The suggested procedures include preappointment patient risk evaluation through a specific questionnaire; frequent hand hygiene; appropriate individual protective equipment; insulation of the oral cavity with a rubber dam after mouth rinses, which are suggested with peroxide 3%; the use of antiretraction handpieces; disinfection after every dental treatment; and appropriate waste management [20]. In Italy there are more than 58,000 dentists (about 1 per 1000 inhabitants), differently distributed at the regional level (Table 2). The management of dental activity can play an important role in limiting the infections. Due to the increasing involvement of a large part of the population in the global epidemic situation in Italy, the present study aimed to assess the knowledge about the new coronavirus, the perception of risk and the clinical management of the risk related to infection during the first month of the Italian epidemic in an online survey of Italian dentists. Moreover, due to the rapid change in the number of infected individuals, a further analysis aimed to evaluate the progressive perception of the risks.

**Table 1:** COVID-19 National trend updated to: March 28th 2020 ^a^

REGIONS	CONFIRMED CASES	RECOVERED CASES	DEATHS	TOTAL CASES	SWAB TESTS
Lombardy	24.509	8.962	5.944	39.415	102.503
Emilia-Romagna	9.964	1.075	1.344	12.383	52.991
Veneto	6.913	655	362	7.930	89.380
Piedmont	6.851	203	617	7.671	21.511
Tuscany	3.511	108	198	3.817	25.613
Marche	2.999	10	364	3.373	9.884
Lazio	2.181	200	124	2.505	27.179
Trentino-Alto Adige	2.163	267	184	2.614	14.729
Liguria	2.086	378	358	2.822	8.177
Campania	1.407	76	109	1.592	10.616
Apulia	1.358	29	71	1.458	11.500
Sicily	1.242	60	57	1.359	13.096
Friuli-Venezia Giulia	1.120	229	87	1.436	12.723
Abruzzi	1.027	30	76	1.133	7.003
Umbria	898	43	28	969	7.028
Sardinia	569	29	26	624	4.225
Calabria	523	11	21	555	7.760
Valle d’Aosta	468	2	41	511	1.380
Basilicata	178	1	3	182	1.421
Molise	98	16	9	123	807
**Total**	**70.065**	**12.384**	**10.023**	**92.472**	**429.526**

^a^**data source**: Italian Civil Protection Department- Ministry of Health

**Table 2:** Distribution of dentists in Italy by Region

Regions	Number of Dentists	Percentages	Population	Inhabitants/Dentist rate number	Dentist number for 1000 inhabitants
Piedmont	4.64	7,33	4.457.335	1.045	1,0
Valle d’Aosta	79	0,14	128.230	1.623	0,6
Lombardy	9.807	16,85	9.917.714	1.011	1,0
Trentino A.A.	827	1,42	1.037.114	1.254	0,8
Veneto	4.464	7,67	4.937.854	1.106	0,9
Friuli V.G.	1.539	2,64	1.235.808	803	1,2
Liguria	2.108	3,62	1.616.788	767	1,3
Emilia-Romagna	4.366	7,50	4.432.418	1.015	1,0
Tuscany	3.948	6,78	3.749.813	950	1,1
Umbria	945	1,62	906.486	959	1,0
Marche	1.839	3,16	1.565.335	851	1,2
Lazio	6.419	11,03	5.728.688	892	1,1
Abruzzi	1.199	2,06	1.342.366	1.120	0,9
Molise	303	0,52	319.780	1.055	0,9
Campania	4.779	8,21	5.834.056	1.221	0,8
Apulia	3.494	6,00	4.091.259	1.171	0,9
Basilicata	418	0,72	587.517	1.406	0,7
Calabria	1.883	3,24	2.011.395	1.068	0,9
Sicily	3.973	6,83	5.051.075	1.271	0,8
Sardinia	1.549	2,66	1.675.411	1.082	0,9
Total	58.203	100,0	60.626.442	1.042	1,0

## Methods

This study used a questionnaire-based survey. The original version of the survey was piloted among a group of dentists to ensure suitability, validity, practicability and interpretation of answers. On the basis of the comments and suggestions obtained, the questionnaire was revised. The questionnaire was developed and forwarded to italian dentists in Italian and translated into English for the presentation of this research (Supplementary file 1). Scientific bases for the development of questions about the new coronavirus came from consultation of the scientific literature available on this subject, more specifically addressed to virologists, specialists, biologists, and general practitioners and epidemiological content that traced the data on coronavirus infection since its first appearance in China [2–10].

Additionally, as a source of scientific information and as an aid for the processing of a part of the questionnaire presented to Italian dentists, circulars issued by the Ministry of Health containing protocols and guidelines aimed at health professionals during the outbreak and informative material disclosed by dental associations were also considered.

The questionnaire was uploaded online to the free survey platform Survio.com (© Survio, Hlinky 70, Brno, Czech Republic) through a specially created user profile. The generated link was shared on professional group and contact networks on the main social channels (LinkedIn, Facebook, and WhatsApp), inviting Italian dentists to share the link with other colleagues in further professional groups to widen the spread of the survey as much as possible. The assurance that those who would respond to the survey were regular dentists was given by the fact that all online professional groups and networks, of which none of the Authors are an administrator, referred to (also for the dissemination of the questionnaire) by regulation are closed and approve registrations only when the registration number of the national professional register is originally provided. The responses were validated only for fully completed questionnaires, in fact the system automatically rejected incomplete questionnaires.

The data collected were absolutely anonymous, and tracing the identity of the subjects was not possible.

A total of 24 open- and close-ended questions were developed (Table 3). Seven questions helped to obtain a profile of the practitioner (age group; sex; type of clinical activity-private, hospital or both; qualification-specialist or not; territorial provenance-specifying region; the number of citizens of their city; and the number of patients treated daily). Six questions were intended to evaluate the direct influence of the coronavirus epidemic on the dentist’s clinical activity (presence or absence of infected cases in their region; questions of patients about coronavirus; patients appearing to be worried or not about possible infections with coronavirus during dental procedures; effective decrease or not in patient appointment number since the coronavirus outbreak onset; adoption of special measures taken during professional activity since the coronavirus emergency started in Italy; and which prevention methods are possibly used).

**Table 3:** Questionnaire and results

QUESTIONS AND ANSWERS	RESULTS	
1. Where do you work? *(Choose an answer)*		
A) In a private dental office	416	77.8%
B) In a public hospital	18	3.4%
C) Both	101	18.9%
2. Which age group do you belong to? *(Choose an answer)*		
A) Up to 35 years	174	32.5%
B) From 36 to 45 years old	146	27.3%
C) From 46 to 60 years old	179	33.5%
D) Over 60	36	6.7%
3. Are you a man or a woman? *(Choose an answer)*		
A) Man	261	48.8%
B) Woman	274	51.2%
4. Are you a specialist? *(Choose an answer)*		
A) Yes, in orthodontics	90	16.8%
B) Yes, in oral surgery	62	11.6%
C) Yes, in paediatric dentistry	9	1.7%
D) No	224	41.9%
E) No, but I predominantly practice a specific branch (e.g. implantprosthesis, endodontics, etc.)	150	28.0%
5. In your region (the one where you practice your professional activity) there have been cases of Coronavirus infection? *(Choose an answer)*		
A) Yes	374	69.9%
B) No	161	30.1%
6. In which region do you exercise your professional activity? (Open answer)		
	Results detailed in Fig.4	
7. How many inhabitants are there in your city? *(Choose an answer)*		
A) Less than 10,000	58	10.8%
B) 10,001 to 330,000	217	40.6%
C) Between 330,001 and 660,000	82	15.3%
D) Between 660,001 and 1 million	46	8,6%
E) Over 1 million	132	24.7%
8. How many patients attend your practice every day? *(Choose an answer)*		
A) Less than 10	101	18.9%
B) No more than 10	172	32.1%
C) Beyond 10	262	49.0%
9. Since the Coronavirus outbreak, have you noticed a decrease in access to your dental office or public hospital you work in? *(Choose an answer)*		
A) Yes	94	17.6%
B) No	270	50.5%
C) Yes, only after the spread of cases in our Country	122	22.8%
D) I don’t know	49	9.2%
10. From a scientific point of view, how much do you think you are informed about Coronavirus? *(Choose an answer)*		
A) Not at all	5	0.9%
B) Little	78	14.6%
C) Enough, I think I’m sucly informed	250	46.7%
D) Very, I think I am properly informed	152	28.4%
E) Very much, my knowledge on the topic is going hand in hand with updates from the international community	50	9.3%
11. How did you get the scientific informations about Coronavirus? *(Choose one or more answers)*		
A) Television, online and/or print newspapers and social media	220	20.8%
B) Institutions (Minister of Health, Italian Government, Order of Physicians, etc.)	398	37.6%
C) Other colleagues	78	7.3%
D) Scientific literature	171	16.1%
E) Professional associations	180	17.0%
F) Other	8	0.8%
G) I am not informed	2	0.2%
12. Coronaviruses are a large family of viruses, known to infect both humans and some animals, whose primary target cells are those epithelial of the respiratory and gastrointestinal tract. How do you judge this statement? *(Choose an answer)*		
A) True	390	72.9%
B) False	88	16.4%
C) I don’t know	57	10.7%
13. What does nCov mean? *(Choose an answer)*		
A) A strain of coronavirus that had not previously been identical in humans	338	63.2%
B) The virus of the common cold	29	5.4%
C) The SARS virus	51	9.5%
D) I don’t know	117	21.9%
14. Is the SARS-Cov-2 virus causing the current coronavirus outbreak? *(Choose an answer)*		
A) No it’s the SARS virus name only	120	22.4%
B) Yes, and it belongs to the same family of acute respiratory syndrome (SARS) virus	90	16.8%
C) Yes, and can also be named 2019-nCov	56	10.5%
D) Answers B and C are correct	236	44.1%
E) none of the previous	33	6.2%
15. What does COVID-19 mean? *(Choose an answer)*		
A) The virus that causes the current outbreak	366	68.4%
B) The disease caused by the new coronavirus	143	26.7%
C) The drug used to treat infected patients	2	0.4%
D) None of the previous answers	24	4.5%
16. What are the most common symptoms in current coronavirus infection? *(Choose an answer)*		
A) Just colds and coughs	2	0.4%
B) Fever, cough and respiratory difficulties	53	9.9%
C) From mild symptoms such as colds, sore throats, fever, muscle aches, coughs to more severe symptoms such as respiratory difficulties and pneumonia	463	86.5%
D) Fever and pneumonia	16	3.0%
E) None of the above answers	1	0.2%
17. How is the new Coronavirus transmitted from person to person? *(Choose an answer)*		
A) Only through saliva	3	0.6%
B) Through saliva, coughing, sneezing, contaminated hands	169	31.6%
C) Through direct personal contact with infected people	36	6.7%
D) None of the previous answers	1	0.2%
E) Options B and C are correct	326	60.9%
18. Are you aware of the existence of a free online course on Coronavirus available to all medical and dental operators promoted by Fnomceo (National Federation of Surgeons and Dentists)? *(Choose an answer)*		
A) Yes and I’ve already done it	31	5.8%
B) Yes, I will	142	26.5%
C) Yes, but I don’t think I do	24	4.5%
D) No, I didn’t know it now and I will	284	53.1%
E) No, I didn’t know but I don’t think I do	54	10.1%
19. Did your patients ask you questions about Coronavirus? *(Choose an answer)*		
A) Yes	349	65.2%
B) No	186	34.8%
20. Do patients seem concerned about the possibility of receiving dental visits/treatments safely? *(Choose an answer)*		
A) Yes	207	38.7%
B) No	328	61.3%
21. Since the spread of Coronavirus in our Country have you taken precautions or taken special measures during the course of the professional activity? *(Choose an answer)*		
A) Yes	369	69.0%
B) No	166	31.0%
22. Which of these prevention methods are you possibly adopting? *(Choose one or more answers)*		
A) Air exchange always between patients and periodically also in the waiting room	177	14.9%
B) In the history include informations about symptoms compatible with infection or recent trips to areas affected by contagion or frequenting with people from those areas (recommended by phone)	118	10.0%
C) Constant use of IPR (individual protective devices) by all dental office/hospital staff	218	18.4%
D) Frequent hand and cleaning of the contact surfaces (e.g. handles or buttons)	234	19.7%
E) Alcohol disinfectant available to patients and carers for hand cleaning at the entrance	115	9.7%
F) All previous	300	25.3%
G) None of the previous	14	1.2%
H) Other	9	0.8%
23. How concerned are you about the spread of Coronavirus infection in our country? *(Choose an answer)*		
A) Not at all	8	1.5%
B) Little	125	23.4%
C) Enough	270	50.5%
D) Very	89	16.6%
E) Very much	43	8.0%
24. One last question... Do you think that dental activity can be considered safe and free from the risk of contagion and spread of the virus for operators and patients? *(Choose an answer)*		
A) Yes	67	12.5%
B) No	468	87.5%

The remaining eleven questions aimed to assess the level of scientific knowledge on coronavirus from a qualitative point of view and the dentist’s perception of the problem related to this emergency in dental clinical practice.

Each respondent to the questionnaire corresponded to a form with all answers provided. The subjects were anonymous and were marked only with a number that reflected the chronological order of compilation. The form showed the day and time when the questionnaire was completed.

The project did not need formal ethical approval since it collected general opinions that do not contain clinical data and neither personal data. According to the current Regulation of the Ethics Committee of the Higher Institute of Health (Istituto Superiore di Sanità), the ethical aspects that need evaluation, approval and monitoring of trial protocols relate to epidemiological, evaluation and medical-social projects that require the collection of personal data. According to the National Data Protection Authority (Garante per la Protezione dei Dati Personali), “personal data” are first and last name, images, tax code, IP address and license plate number. The compilation of the survey was anonymously carried out on a voluntary basis without the possibility to trace the identity of the subjects, as the system does not store even the IP addresses of the users accessing the link. Before the start of the survey, informed consent was presented on the main page; the participant had to agree (by checking a box) that their anonymously provided answers could be used in this research for scientific purposes.

For statistical examination of the data, the online platform automatically generated descriptive statistical analysis on the main page; the analysis could therefore be downloaded as an Excel or SPSS spreadsheet for further statistical analysis. In this study, descriptive statistical analysis was carried out. Several measures of association was performed including, the common Pearson’s χ2, the Likelihood-Ratio χ2, Cramér’s V, Fisher’s exact test, Goodman and Kruskal’s γ, and Kendall’s τb. The level of statistical significance was set at 0.05. The software used is STATA 15.1 (StataCorp LLC, TX, USA).

The Pearson’s and Likelihood-Ratio χ2 test for the independence of the rows and columns. The null hypothesis (H0) is that there is no relationship. To reject this we need a *P* < 0.05 (at 95% confidence).

Cramér’s V is a measure of association between two nominal variables. It goes from 0 to 1, where 1 indicates strong association. γ and τb are measures of association between two ordinal variables (both have to be in the same direction, i.e. negative to positive, low to high). Both go from − 1 to 1. Negative shows inverse relationship, closer to 1 a strong relationship. γ is recommended when there are lots of ties in the data. τb is recommended for square tables.

Fisher’s exact test is used when there are very few cases in the cells (usually less than 5, with an overall frequency of less than 20%). It tests the relationship between two variables. The null is that variables are independent [21–25].

## Results

The survey was online for 3 weeks from 23 February 2020 to 15 March 2020. The link received 795 visits, but only 535 dentists responded to the survey by completing it. The results of the descriptive statistics were collected in Table 3.

Most dentists carried out their professional activity in a private practice (77.8%).The age group of up to 35 years old (yo) and the group between 46 and 60 yo were the most represented (respectively, between 32.5 and 33.5%). The distribution between the two sexes was equivalent (48.8% males and 51.2% females).

Over 41.9% of dentists were General Dentists, 28% are Dentists without a recognized dental specialty (Italian Universities provide 3 years of postgraduate programs in Orthodontics, Oral Surgery and Pediatric Dentistry. They are the only recognized Dental Specialties) and 30.8% are Dental Specialists (16.8% were orthodontists, approximately 12% were oral surgeons, and just over 2% were specialists in pediatric dentistry).

**Fig. 4:**
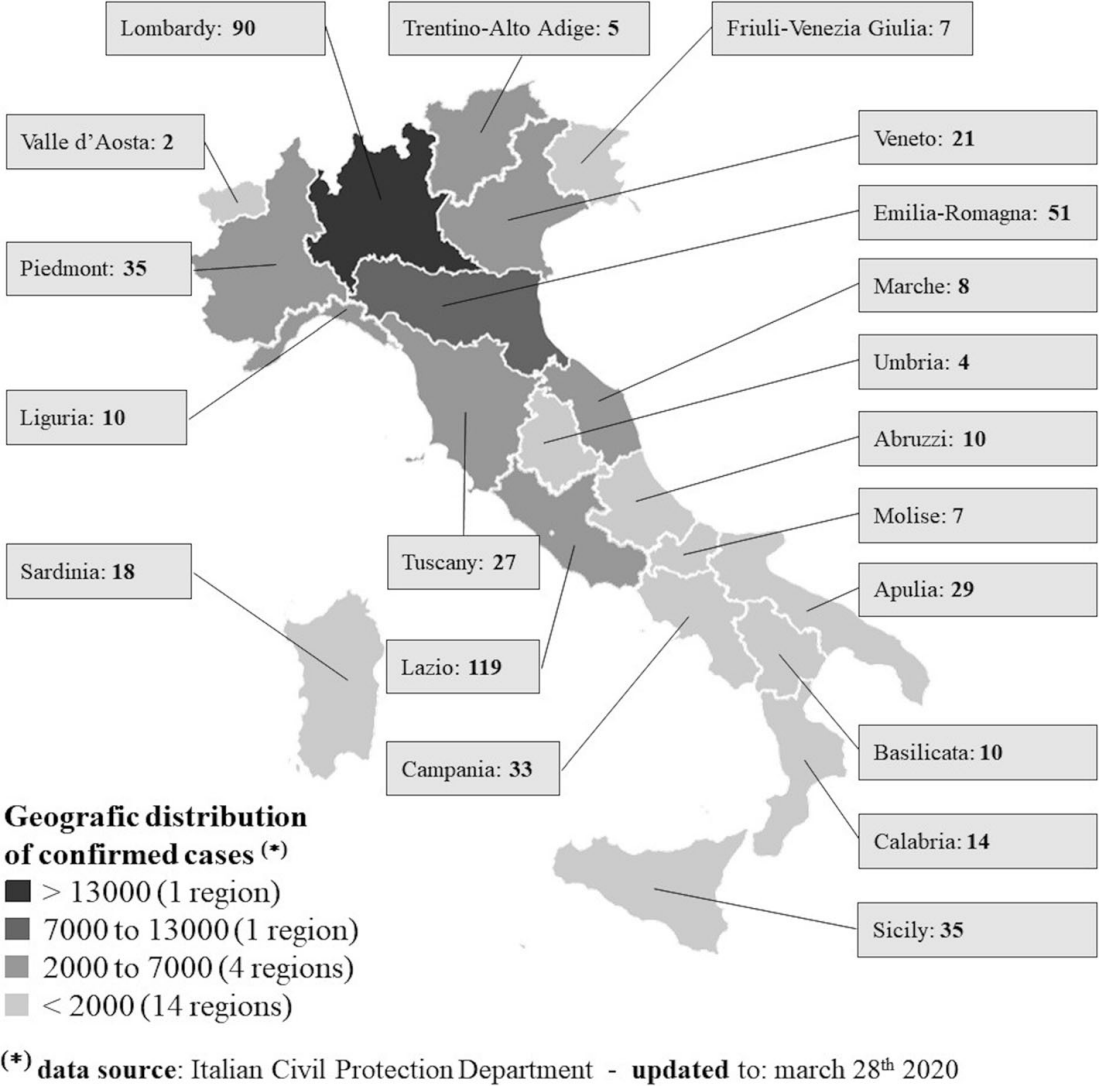
The rectangles with the names of the different regions of Italy are accompanied by the number of dentists who participated in the survey. The color scale distinguishes the different distribution of confirmed cases (data source: Italian Civil Protection, updated to March 28th 2020)

The answers related to the geographical location of the workplace mapped across the whole country, representing Italy from north to south and including the larger islands (Sicily and Sardinia) (Fig. 4).

Most of the respondents (40.6%) were from moderately or highly populated cities. Fourty 9 % of the dentists who participated in the survey treat more than 10 patients per day. Almost 70 % of dentists completed the questionnaire when there were positive cases in their region of SARS-CoV-2 infection.

Fifty percent of respondents did not notice a decrease in visits since the outbreak spread. More than 65% of patients asked questions about coronavirus to their dentist. According to the clinicians who participated in the study, the majority of patients (more than 61%) would not be worried about getting coronavirus infection during dental treatment.

Almost 47% of dentists said they were fairly informed about coronavirus. Despite of the self-estimated knowledge about the infection, answers to the following questions assessing knowledge on the subject revealed a different reality. Most respondents obtained scientific information about coronavirus through Italian institutions (37.6%); television, newspapers and social media (20.8%); professional associations (17%); scientific literature (l6.1%); and other colleagues (7.3%). Only a very small percentage (0.8%) specified other channels of information or that they were not fully informed (0.2%).

Almost 73% correctly answered the questions about the definition of coronavirus, the 63.2% correctly answered about nCoV and 44.1% about SARS-CoV-2. Most respondents, on the other hand, incorrectly answered the question on the definition of COVID-19 (almost 69%).

Almost 87% of the subjects were very clear about the types of possible symptoms that accompany the infection, and in 60.9% of cases, they correctly indicated how the new coronavirus is transmitted from person to person.

However, the 63.2% of dentists knew that the National Federation of Surgeons and Dentists (Federazione Nazionale dei Medici Chirurghi ed. Odontoiatri- FNOMCEO) has provided healthcare professionals with a free online course to disseminate useful information about the virus.

Sixty nine percent of dentists who completed the questionnaire had taken safety and prevention measures against workplace infection since coronavirus spread. Almost 26% of them had taken all the recommended safety measures (telephone history collection, increased frequency of washing hands and environmental surfaces, and personal protective equipment such as gloves, disposable gowns and facemasks with adequate filters).

Fifty point 5 % of respondents were concerned ‘enough’ about the spread of infection in Italy. Overall, almost 88% of dentists who took part in the survey considered the dental profession neither safe nor free from the risk of contagion for both patients and healthcare professionals.

The measures of association results were collected in Tables 4, 5 and 6. In regards to the sex (Table 4), both *χ*^*2*^ test statistics show a significance level < 0.05 for quality of information (question number 10), level of information related to questions 12 and 17 and for risk perception related to question 23; so we can safely assume that some differences exist between groups. Therefore, we conclude that there is evidence of a statistically significant difference between male and female on these variables. We can confidently reject the null hypothesis that these two variables are statistically independent in that population. In other words, we can conclude that there is some relationship between sex and each of these four variables. In fact, for these variables the Cramér’s *V* values are > 0.13, which indicates a non-negligible association. Moreover, the Goodman and Kruskal’s lambda for the relationship between sex and level of information related to question number 12, and sex and risk perception related to question 23 is > 0.22, in line with previous results. All these findings are confirmed by Fisher’s exact test results, since in these four cases the hypothesis of variables’ independence is rejected, and we conclude that there is some kind of relationship between variables.

**Table 4:** Measures of association -gender

	Pearson ***χ***^**2**^ test	LR ***χ***^**2**^ test	Cramér’s ***V***	Goodman-Kruskal’s ***γ***	Kendall’s ***τ***_***b***_	Fisher’s exact test
1. Gender- QUALITY OF INFORMATION(10)	9.6496** **(0.047)**	9.8368** **(0.043)**	0.1343	−0.0319 (0.069)	− 0.0185 (0.040)	(0.044)
2. **Gender-** **LEVEL OF INFORMATION(18)**	**3.8129** **(0.432)**	**3.8238** **(0.430)**	**0.0844**	**− 0.0871** **(0.071)**	**−0.0491** **(0.040)**	**(0.434)**
3. Gender- LEVEL OF INFORMATION(12)	9.2567*** **(0.010)**	9.3697*** **(0.009)**	0.1315	0.2681 (0.086)	0.1254 (0.041)	(0.010)
4. Gender- LEVEL OF INFORMATION(13)	1.5626 (0.668)	1.5670 (0.667)	0.0540	−0.0029 (0.079)	−0.0015 (0.041)	(0.674)
5. Gender- LEVEL OF INFORMATION(14)	0.7400 (0.946)	0.7403 (0.946)	0.0372	0.0243 (0.066)	0.0145 (0.040)	(0.947)
6. Gender- LEVEL OF INFORMATION(15)	2.8786 (0.411)	3.6511 (0.302)	0.0734	0.0664 (0.088)	0.0318 (0.042)	(0.492)
7. Gender- LEVEL OF INFORMATION(16)	5.1846 (0.269)	5.6270 (0.229)	0.0984	0.1794 (0.121)	0.0623 (0.042)	(0.190)
8. Gender- LEVEL OF INFORMATION(17)	10.5200** **(0.033)**	10.9648** **(0.027)**	0.1402	−0.1012 (0.081)	−0.0523 (0.042)	(0.015)
9. Gender- CORRECT RISK MANAGEMENT(22)	1.7211 (0.190)	1.7214 (0.190)	−0.0567	−0.1221 (0.092)	− 0.0567 (0.043)	(0.192)
10. Gender- RISK PERCEPTION(23)	24.9374*** **(0.000)**	25.7561*** **(0.000)**	0.2159	0.2272 (0.067)	0.1322 (0.040)	(0.000)
11. Gender- RISK PERCEPTION(24)	1.2709 (0.260)	1.2717 (0.259)	0.0487	0.1466 (0.128)	0.0487 (0.043)	(0.296)

Notes: unequal variances assumed, after some checks. *P*-Values in parentheses. For Goodman-Kruskal’s *γ* and Kendall’s *τ*-b the Asymptotic Standard Errors (ASE) are reported. *p* < 0.10, *p* < 0.05, *p* < 0.01

**Table 5:** Measures of association- age

	Pearson ***χ***^**2**^ test	LR ***χ***^**2**^ test	Cramér’s ***V***	Goodman-Kruskal’s ***γ***	Kendall’s ***τ***_***b***_	Fisher’s exact test
12. Age- QUALITY OF INFORMATION(10)	39.3684*** **(0.000)**	38.2612*** **(0.000)**	0.1566	−0.0835 (0.055)	− 0.0580 (0.038)	
13. Age- LEVEL OF INFORMATION(18)	17.6918 (0.125)	18.1606 (0.111)	0.1050	−0.0814 (0.058)	−0.0547 (0.039)	
14. Age- LEVEL OF INFORMATION(12)	1.5170 (0.958)	1.5206 (0.958)	0.0377	−0.0131 (0.070)	−0.0072 (0.039)	(0.947)
15. Age- LEVEL OF INFORMATION(13)	15.2818* **(0.083)**	15.6249* **(0.075)**	0.0976	−0.2079 (0.060)	−0.1289 (0.038)	
16. Age- LEVEL OF INFORMATION(14)	20.0687* **(0.066)**	19.5222* **(0.077)**	0.1118	−0.1332 (0.051)	−0.0948 (0.036)	
17. Age- LEVEL OF INFORMATION(15)	7.0758 (0.629)	7.9172 (0.543)	0.0664	0.1031 (0.068)	0.0588 (0.039)	(0.488)
18. Age- LEVEL OF INFORMATION(16)	8.8334 (0.717)	10.1643 (0.602)	0.0742	0.0679 (0.093)	0.0280 (0.038)	(0.545)
19. Age- LEVEL OF INFORMATION(17)	19.3533* **(0.080)**	19.2796* **(0.082)**	0.1098	0.0726 (0.063)	0.0446 (0.039)	
20. Age- CORRECT RISK MANAGEMENT(22)	1.1150 (0.773)	1.1188 (0.773)	0.0457	−0.0303 (0.072)	−0.0166 (0.040)	(0.777)
21. Age- RISK PERCEPTION(23)	13.6528 (0.552)	13.6826 (0.550)	0.0922	−0.0193 (0.056)	−0.0132 (0.038)	
22. Age- RISK PERCEPTION(24)	6.8839* **(0.076)**	5.9705 (0.113)	0.1134	0.1004 (0.101)	0.0402 (0.041)	(0.093)

Notes: unequal variances assumed, after some checks. *P*-Values in parentheses. For Goodman-Kruskal’s *γ* and Kendall’s *τ*-b the Asymptotic Standard Errors (ASE) are reported. *p* < 0.10, *p* < 0.05, *p* < 0.01

**Table 6:** Measures of association- region

	Pearson ***χ***^**2**^ test	LR ***χ***^**2**^ test	Cramér’s ***V***	Goodman-Kruskal’s ***γ***	Kendall’s ***τ***_***b***_
23. Region- QUALITY OF INFORMATION(10)	63.4819 (0.912)	68.3577 (0.820)	0.1722	−0.0254 (0.044)	−0.0197 (0.034)
24. Region- LEVEL OF INFORMATION(18)	95.6957 (0.111)	99.0171* **(0.073)**	0.2115	−0.0046 (0.047)	−0.0035 (0.035)
25. Region- LEVEL OF INFORMATION(12)	43.3894 (0.329)	47.8005 (0.186)	0.2014	−0.0396 (0.055)	−0.0246 (0.034)
26. Region- LEVEL OF INFORMATION(13)	88.8741*** **(0.009)**	97.3943*** **(0.002)**	0.2353	−0.0473 (0.049)	−0.0329 (0.034)
27. Region- LEVEL OF INFORMATION(14)	114.5570*** **(0.007)**	104.4948** **(0.034)**	0.2314	0.0086 (0.044)	0.0068 (0.035)
28. Region- LEVEL OF INFORMATION(15)	77.3373* **(0.065)**	50.7542 (0.797)	0.2195	−0.0732 0.058	−0.0472 (0.037)
29. Region- LEVEL OF INFORMATION(16)	77.2407 (0.567)	64.3017 (0.900)	0.1900	−0.0493 (0.081)	−0.0229 (0.038)
30. Region- LEVEL OF INFORMATION(17)	76.1563 (0.601)	54.2159 (0.988)	0.1886	−0.0450 (0.053)	−0.0309 (0.037)
31. Region- CORRECT RISK MANAGEMENT(22)	42.1485*** **(0.003)**	44.5656*** **(0.001)**	0.2807	0.0276 (0.061)	0.0172 (0.038)
32. Region- RISK PERCEPTION(23)	66.2305 (0.996)	68.6691 (0.993)	0.1574	0.0420 (0.045)	0.0322 (0.035)
33. Region- RISK PERCEPTION(24)	23.0833 (0.285)	22.4473 (0.317)	0.2077	0.0816 (0.089)	0.0367 (0.040)

Notes: unequal variances assumed, after some checks. *P*-Values in parentheses. For Goodman-Kruskal’s *γ* and Kendall’s *τ*-b the Asymptotic Standard Errors (ASE) are reported. *p* < 0.10, *p* < 0.05, *p* < 0.01

Concerning the age (Table 5), the Pearson and Likelihood-Ratio *χ*^*2*^ tests present a *P*-Value < 0.05 only for the relationship with the variable quality of information (question number 10). We reject the null hypothesis of no association at conventional level of statistical significance, because it emerges a dependence of the rows and columns. Thus, in this case we can conclude that some differences emerge between groups. Moreover, in this case Cramér’s *V* is > 0.15: there is a small but statistically significant association between these variables.

If we consider the region (Table 6), the Pearson and LR *χ*^*2*^ tests show a *P*-Value < 0.05 for level of information related to questions number 13 and 14, and correct risk management related to question number 22; therefore, we conclude that some relationship exists between region and each of these three variables. Here, the Cramér’s *V* are > 0.23, which indicate a statistically significant association.

## Discussion

Since SARS-CoV-2 can be transmitted from person to person by droplets, contact and through saliva, dental patients and dentists and their coworkers can be easily exposed to novel coronavirus infections [19, 26, 27]. In the period of contagion outbreak from the new coronavirus, information about the virus has become increasingly the subject of attention of the media, such as television, the internet, and social channels. However, it was only when the first cases began to register in Italy that professional associations and dental professionals began to take a deeper interest in the problem. Dental professionals had to refer only to the official communication of the ministry, whose law decrees lacked specific references to the dental profession.

Male dentists believed to be very well informed about Coronavirus unlike female colleagues who had a more cautious opinion on their knowledge of the subject (Pearson χ2 test 9.6496- p 0.047; LR χ2 test 9.8368- p 0.043). Dentists between the ages of 46–60 believe they were well informed compared to younger colleagues who judged sufficient their knowledge (Pearson χ2 test 39.3684- p 0.000; LR χ2 test, 38.2612 –p 0.000). Male dentists showed to have a significantly clearer idea of the taxonomic characteristics of the virus (Pearson χ2 test 9.2567- p 0.010; LR χ2 test 9.3697- p 0.009). Most were aware of the main features of coronaviruses but confused the term COVID-19 with the virus itself (68.4%). The definition of COVID-19 was provided more correctly by the dentists of Lazio, Lombardy, Emilia-Romagna and Sicily but the same regions, with the exception of Sicily, reported the greatest number of incorrect answers (which overall exceeded the correct ones) and attributed to this term the meaning of “virus that causes the disease”(Pearson χ2 test 77.3373- p 0.065). The question containing the request to identify the correct definition of COVID-19 was absolutely, among all the questions in the questionnaire provided with the aim of assessing scientific knowledge on the subject, the one for which the largest number of wrong answers were recorded.

In addition, most believed that the term SARS-CoV 2 is not related to the new coronavirus but is rather the name of the SARS virus that caused an epidemic in 2002–2003 (22.4%). Dentists aged between 36 and 45 have identified the correct answer in a significantly higher percentage than younger and older colleagues (Pearson χ2 test 20.0687 - p 0.066; LR χ2 test, 19.5222 -p 0.077). Also on this definition, the dentists of Lazio and Lombardy were those significantly better informed, followed by their colleagues from Emilia-Romagna and Campania (Pearson χ2 114.5570- p 0.007; LR χ2 test 104.4948- p 0.034).

The most informed dentists on the possibility to access a free online course on the new Coronavirus promoted by the FNOMCEO (National Federation of Surgeons and Dentists) were those from Lazio, followed by those from Lombardy, Sicily and Tuscany (LR χ2 test 99.0171- p 0.073).

Quite important for the population and for the spread of epidemics is the preventive approach of dentists. For the possibilities of transmission from person to person, most are properly informed (60.9%). Female dentists were better informed on this aspect (Pearson χ2 test 10.5200- p 0.033; LR χ2 test 10.9648- p 0.027). Compared to age, younger dentists were significantly better informed about the transmission routes of the virus than other age groups (Pearson χ2 test, 19.3533- p 0.080; LR χ2 test 19.2796- p 0.082).

More than 87% of respondents to the survey were aware of the wide variety of symptoms with which the infection can occur, which is encouraging because it means that a diagnostic suspicion and a report to the authorities regulating the execution of swab tests of potentially infected individuals can also be appropriately carried out by a dentist. It is important to consider that transmission may occur through asymptomatic patients and that symptoms when COVID-19 is present can also be mild and confused with a simple cold or flu [28]. Its manifestation does not always culminate with severe symptomatology accompanied by respiratory failure up to interstitial pneumonia. The asymptomatic incubation period takes approximately 1–14 days, and in these days, persons without symptoms can spread the virus. For this reason, it is important to add to the information required of the patient in the medical history, the report of a possible contact with infected or potentially infected people or of trips to the areas where the infection has spread. Many dentists (10%) who responded to the survey chose to ask this question of their patients, judging it to be important. At the time of the virus’s main spread, it was recommended to perform a telephone triage even before seeing the patient to assess whether to visit or to postpone the appointment.

At the time of the survey, performed between February 23rd and March 15th, just over 50% of dentists did not notice a reduction in the number of visits despite the spread of the virus. It must be specified that after two weeks from the start of this research many work activities were suspended by the government by extraordinary decree, but the clinical dental activity was allowed only for the management of emergencies provided by dentists equipped with adequate personal protective equipment (PPE). The definition of “adequate PPE” for dentists is a matter of debate because above all the surgical masks used routinely by dentists would not have sufficient filters to protect from infection. The use of facemasks with ffp2 or ffp3 filters, highly protective than the surgical ones, does not seem to be considered necessary for routine dental activity, even if this has not been clearly said nor denied.

The absence of a sample calculation and the methodology used in the dissemination of the survey represent limitations in this research. Anyway the distribution of the respondents in the national territory was quite homogenous (proportionate to the extension of the individual regional territories) and the greatest proportion came from medium-large cities. Although 65.2% of dentists said that patients have asked questions about coronavirus, they agree that patients feeling worried about contracting the infection through dental care has not emerged (61.3%). Most of the dentists (69%) adopted additional preventive practical measures, a sign of a growing and widespread awareness (87.5%) of the risk of contributing to the spread of contagion through dental activity. There were regions where the number of dentists who claimed to have taken additional contagion prevention measures during their clinical activity was significantly higher than others. This was especially true for regions such as Lazio, Lombardy, Emilia Romagna and Campania. It is interesting to note that regions such as Veneto which since the beginning of the spread of the virus in Italy has been one of the first regions and among the most affected had not the same attitude (Pearson χ2 test, 42.1485- p 0.003; LR χ2 test 44.5656- p 0.001).

The female gender appeared significantly more concerned than the male gender about the spread of Coronavirus infection (Pearson χ2 test 24.9374- p 0.000; LR χ2 test 25.7561, p 0.000). Dentists belonging to the younger age groups were found to be much more convinced than their older colleagues that this epidemic has future repercussions on the dental profession as it is not without risk (Pearson χ2 test, 6.8839- p 0.076).

Dental treatment procedures always involve close contact with the patient, and this setup does not allow the maintenance of an adequate safe distance. It is extremely important that dentists equip themselves with appropriate individual safety devices (masks, gloves, protective goggles, hair caps and shirts). A recent article in the New York Times, referring to the database “O’NET” used by the Department of Labor to describe the various physical aspects of different professions, highlighted that the occupational categories in which you come into physical contact with others are those where the risk of COVID-19 is highest. Dentists are at the top of the ranking for work-related risk [29]. In this survey, dentists affirmed the constant use of these safety devices as prescribed by the Italian medical guidelines of safety in workplaces [30]. When aerosol procedures are carried out, the presence of saliva and blood increases the spread of germs, bacteria and viruses. Ensuring a change of air in the workplace and in the waiting room is a simple but important measure chosen by 14.9% of dentists in this survey. This measure should always be adopted by dentists and not only in this situation. Equally essential is to wash hands more frequently and disinfect them with alcohol-based solutions. This provision should also be encouraged for patients before entering the operating dental unit. These recommendations, together with those of not shaking hands with anyone, were accepted by 9.7 and 19.7% of respondents, respectively. The data that emerged on the cleansing measures also include the cleaning of the clinical contact surfaces, such as buttons, handles and work surfaces. Thorough cleaning has proven to be a mandatory and indispensable choice for prevention, as it is proven that the coronavirus family, including SARS-CoV-2, can survive on plastic, metal and glass surfaces for up to 9 days and can be efficiently deactivated through disinfection procedures with 62–71% ethanol, 0.5% hydrogen peroxide or 0.1% sodium hypochlorite within 1 min. The use of 0.05–0.2% benzalkonium chloride or 0.02% chlorhexidine digluconate does not have the same effectiveness [31]. It must be said that the majority of dentists paid great attention to the measures to be taken; indeed, 25.3% of them said they had adopted all the preventive measures listed so far.

## Conclusions

This is the most severe epidemic that has hit Italy in the past 100 years, and it will probably be one of the most severe viral pandemics of modern times. As no specific therapies are available at the moment for the new coronavirus, prevention and early containment of further spread can be crucial to control the pandemic. For this reason, dentists, similar to other medical practitioners, aware of the risk associated with carrying out their professional activity, at this moment limited to the management of dental emergencies only, have the responsibility in this situation to know the characteristics of the virus through precise and accurate information and to assume a careful and proactive attitude for the protection of their patients and of their entire community, working in the containment of this social emergency even if not directly involved in the treatment of affected patients.

Dentists at this time, however, should only work if they have the individual protective equipment recommended to high-risk healthcare workers [32, 33]. After the pandemic emergency when people’s professional activities and lives can slowly return to normal, the experience and the not-quite-finished risk of a recurrence of new cases of infection will require that dentists also follow new health safety protocols whose definition will be necessary.

## Supplementary information

**Additional file 1.** “Questionnaire” contains the English version of the questionnaire realized for the survey in this research.

## Data Availability

The datasets used and/or analysed during the current study available from the corresponding author on reasonable request.

## Abbreviations

WHO: World Health Organization
PPE: Personal Protection Equipment
