# Supplementary material for: Coronavirus (COVID-19) in Italy: knowledge, management of patients and clinical experience of Italian dentists during the spread of contagion
Source: BMC Oral Health. 2020 Jul 10;20:200. doi: 10.1186/s12903-020-01187-3 (PMC7349471; doi:10.1186/s12903-020-01187-3)
Supplement: Supplementary file 1 — Additional file 1. “Questionnaire” contains the English version of the questionnaire realized for the survey in this research. [file 12903_2020_1187_MOESM1_ESM.docx]

QUESTIONNAIRE

1. Where do you work? (Choose an answer)

A) In a private dental office

B) In a public hospital

C) Both

2. Which age group do you belong to? (Choose an answer)

A) Up to 35 years

B) From 36 to 45 years old

C) From 46 to 60 years old

D) Over 60

3. Are you a man or a woman? (Choose an answer)

A) Man

B) Woman

4. Are you a specialist? (Choose an answer)

A) Yes, in orthodontics

B) Yes, in oral surgery

C) Yes, in paediatric dentistry

D) No

E) No, but I predominantly practice a specific branch (e.g. implantprosthesis, endodontics, etc.)

5. In your region (the one where you practice your professional activity) there

have been cases of Coronavirus infection? (Choose an answer)

A) Yes

B) No

6. In which region do you exercise your professional activity?

(Open answer)

7. How many inhabitants are there in your city? (Choose an answer)

A) Less than 10000

B) 10001 to 330000

C) Between 330001 and 660000

D) Between 660001 and 1 million

E) Over 1 million

8. How many patients attend your practice every day? (Choose an answer)

A) Less than 10

B) No more than 10

C) Beyond 10

9. Since the Coronavirus outbreak, have you noticed a decrease in access to your dental office

or public hospital you work in? (Choose an answer)

A) Yes

B) No

C) Yes, only after the spread of cases in our Country

D) I don't know

10. From a scientific point of view, how much do you think you are informed

about Coronavirus? (Choose an answer)

A) Not at all

B) Little

C) Enough, I think I'm sucly informed

D) Very, I think I am properly informed

E) Very much, my knowledge on the topic is going hand in hand with updates from the international community

11. How did you get the scientific informations about Coronavirus?

(Choose one or more answers)

A) Television, online and/or print newspapers and social media

B) Institutions (Minister of Health, Italian Government, Order of Physicians, etc.)

C) Other colleagues

D) Scientific literature

E) Professional associations

F) Other

G) I am not informed

12. Coronaviruses are a large family of viruses, known to infect both humans and some animals,

whose primary target cells are those epithelial of the respiratory and gastrointestinal tract.

How do you judge this statement? (Choose an answer)

A) True

B) False

C) I don't know

13. What does nCov mean? (Choose an answer)

A) A strain of coronavirus that had not previously been identical in humans

B) The virus of the common cold

C) The SARS virus

D) I don't know

14. Is the SARS-Cov-2 virus causing the current coronavirus outbreak? (Choose an answer)

A) No it’s the SARS virus name only

B) Yes, and it belongs to the same family of acute respiratory syndrome (SARS) virus

C) Yes, and can also be named 2019-nCov

D) Answers B and C are correct

E) none of the previous

15. What does COVID-19 mean? (Choose an answer)

A) The virus that causes the current outbreak

B) The disease caused by the new coronavirus

C) The drug used to treat infected patients

D) None of the previous answers

16. What are the most common symptoms in current coronavirus infection? (Choose an answer)

A) Just colds and coughs

B) Fever, cough and respiratory difficulties

C) From mild symptoms such as colds, sore throats, fever, muscle aches, coughs to more severe symptoms such as respiratory difficulties and pneumonia

D) Fever and pneumonia

E) None of the above answers

17. How is the new Coronavirus transmitted from person to person? (Choose an answer)

A) Only through saliva

B) Through saliva, coughing, sneezing, contaminated hands

C) Through direct personal contact with infected people

D) None of the previous answers

E) Options B and C are correct

18. Are you aware of the existence of a free online course on Coronavirus available

to all medical and dental operators promoted by Fnomceo

(National Federation of Surgeons and Dentists)? (Choose an answer)

A) Yes and I've already done it

B) Yes, I will

C) Yes, but I don't think I do

D) No, I didn't know it now and I will

E) No, I didn't know but I don't think I do

19. Did your patients ask you questions about Coronavirus? (Choose an answer)

A) Yes

B) No

20. Do patients seem concerned about the possibility of receiving

dental visits/treatments safely? (Choose an answer)

A) Yes

B) No

21. Since the spread of Coronavirus in our Country have you taken precautions

or taken special measures during the course of the professional activity?

(Choose an answer)

A) Yes

B) No

22. Which of these prevention methods are you possibly adopting?

(Choose one or more answers)

A) Air exchange always between patients and periodically also in the waiting room

B) In the history include informations about symptoms compatible with infection or recent trips to areas affected by contagion or frequenting with people from those areas (recommended by phone)

C) Constant use of IPR (individual protective devices) by all dental office/hospital staff

D) Frequent hand and cleaning of the contact surfaces (e.g. handles or buttons)

E) Alcohol disinfectant available to patients and carers for hand cleaning at the entrance

F) All previous

G) None of the previous

H) Other

23. How concerned are you about the spread of Coronavirus infection

in our country? (Choose an answer)

A) Not at all

B) Little

C) Enough

D) Very

E) Very much

24. One last question... Do you think that dental activity can be considered safe and free from the

risk of contagion and spread of the virus for operators and patients? (Choose an answer)

A) Yes

B) No
